# Association of psychological distress and current cigarette smoking among Native Hawaiian and Pacific Islander adults and compared to adults from other racial/ethnic groups: Data from the National Health Interview Survey, 2014

**DOI:** 10.1016/j.pmedr.2021.101660

**Published:** 2021-12-09

**Authors:** Marie-Rachelle Narcisse, Sumit K. Shah, Holly C. Felix, Page D. Dobbs, Pearl A. McElfish

**Affiliations:** a University of Arkansas for Medical Sciences Northwest, College of Medicine, 1125 N. College Avenue, Fayetteville, AR 72703, USA; b University of Arkansas for Medical Sciences Northwest, Office of Community Health and Research, 1125 N. College Avenue, Fayetteville, AR 72703, USA; c University of Arkansas for Medical Sciences, Fay W. Boozman College of Public Health, 4301 W. Markham St., Little Rock, AR 72205, USA; d University of Arkansas, Department of Health, Human Performance and Recreation, 751 W. Maple St., Fayetteville, AR 72701, USA

**Keywords:** Cigarette smoking, Psychological distress, Native Hawaiian and Pacific Islander adults, Racial/ethnic disparities, National Health Interview Survey

## Abstract

Cigarette smoking is the leading cause of preventable deaths worldwide. Research has documented an association between psychological distress and smoking among certain racial/ethnic groups but has not examined this association among Native Hawaiian and Pacific Islander (NHPI) adults. Data from the 2014 general and the NHPI-specific National Health Interview Surveys were analyzed at the University of Arkansas for Medical Sciences Regional Campus (Fayetteville, AR) in April 2021 to determine the association between current cigarette smoking and levels of psychological distress among NHPI adults and to assess the difference in the magnitude of that association among NHPI adults compared to adults of other racial/ethnic groups in the United States. The final analytic sample (n = 34,782) included 1,916 NHPI, 20,430 White, 4,725 Black, 2,001 Asian, and 5,710 Hispanic adults. A significant association between current cigarette smoking and psychological distress levels was found among NHPI adults as well as among adults from other racial/ethnic groups. There was no significant difference in the magnitude of the detected association among NHPI adults compared to the magnitude of the same association detected among adults of other racial/ethnic groups. However, this finding indicates race/ethnicity does not moderate the association between psychological distress and current cigarette smoking. Future studies should explore factors that may further explain the variation in current cigarette smoking within and across all racial/ethnic groups.

## Highlights

• Smoking and distress levels differ significantly by racial/ethnic group.
• Smoking and psychological distress are significantly related among NHPI adults.
• Smoking and psychological distress are also related in other racial/ethnic groups.
• No difference in association magnitude was found between racial/ethnic groups.

## 1 Introduction

Cigarette smoking is the leading cause of preventable deaths and a major public health issue (World Health Organization, 2021). Adults who smoke are estimated to die approximately ten years earlier than those who do not smoke. More than 7 million deaths worldwide and more than 480,000 deaths in the United States (US) are attributable to smoking (Centers for Disease Control and Prevention, 2021). Studies have proven that smoking leads to respiratory tract disorders, cardiovascular disease, strokes, diabetes, and immune system dysfunction (Musk and de Klerk, 2003, National Center for Chronic Disease Prevention and Health Promotion US Office on Smoking and Health, 2014, Centers for Disease Control and Prevention, 2021) as well as cancers affecting almost all major systems of the body (Sasco et al., 2004). Moreover, the provision of medical care attributed to smoking-related medical conditions and loss of productivity due to premature death caused by smoking imposes a substantial financial burden on the healthcare system (Centers for Disease Control and Prevention, 2021).

Smoking rates in the US have declined considerably from 24.1% in 1998 (Centers for Disease Control and Prevention, 2000) to 13.7% in 2018 (Cornelius et al., 2020). Historically, disparities in cigarette smoking have been observed, with adults from most racial/ethnic groups, including Native Hawaiian and Pacific Islander (NHPI) adults, smoking at higher rates than White adults (Caraballo et al., 2008). A few studies have reported cigarette smoking rates for NHPI adults (Mukherjea et al., 2014, Nguyen, 2019, Mattingly et al., 2020), including a 2017 report that showed the 2014 prevalence rate of cigarette smoking among NHPI adults (16.5%) was nearly the same as the rate for the US population (17.0%) (Galinsky et al., 2017).

A number of factors, including socioeconomic status, smoking by parents and peers, attitudes of family and friends, lifestyle habits, and health concerns, have been shown to be related to smoking in general populations (Forman-Hoffman et al., 2017, Garrett et al., 2015, Tyas and Pederson, 1998). In addition, psychological distress, a mental health indicator associated with impairment (Kessler et al., 2002, Kessler et al., 2003, Kim et al., 2012), has also been associated with current cigarette smoking (Forman-Hoffman et al., 2017, Prochaska et al., 2017). As much as 56.4% of the overall cigarette consumption in the US has been attributed to those who experience psychological distress (Talati et al., 2016). Moreover, some studies have found adults with psychological conditions are less likely to maintain a quit attempt when compared to those without psychological conditions (Bandiera et al., 2016, Zvolensky et al., 2015), with stress-induced smoking initiation following a quit attempt documented as a contributing factor (Siegel et al., 2017). No study could be identified in the literature that examined and compared predictors – including psychological distress – of current cigarette smoking among representative samples of different racial/ethnic groups including the NHPI population. However, a few studies have examined predictors of smoking cigarettes within the NHPI population as a whole and within certain NHPI subgroups residing in the US or US territories (e.g., Samoans) and found age, sex, US state of residence, income, education, marital status, and level of acculturation to be significant predictors (Bitton et al., 2010, Mishra et al., 2005, Maskarinec, 2005). Additionally, increased social support was associated with lower smoking rates among Native Hawaiians (Pokhrel et al., 2016).

Despite a growing NHPI population in the US (Hixon and Jones, 2012), this population has been underrepresented in health-related research (Ro and Yee, 2010, Wu and Bakos, 2017). Moreover, the health status and health behaviors of the NHPI population have been historically aggregated with data from the Asian American population in past research (Chae et al., 2006, Kaholokula et al., 2006) even though the US Census Bureau split the Asian American/Pacific Islander racial category into two separate race categories in 1997. The failure to include adequate representation of NHPI participants in health research masks potential racial disparities and health inequities. To help address the lack of health-related data specific to the NHPI population, the National Center for Health Statistics conducted the 2014 US-based National Health Interview Survey (NHIS) with the NHPI population (NHPI-NHIS), which allowed for the exploration of health status and behavior of NHPI adults not capable with prior datasets.

### 1.1 Study aims

Our first aim was to assess smoking prevalence and psychological distress levels among NHPI adults and among those of other racial/ethnic groups and then compare those descriptive results between the NHPI adults and adults of other racial/ethnic groups. Our second aim was to examine the association between psychological distress level and current cigarette smoking for each racial/ethnic group and then compare the results between the NHPI group and the other racial/ethnic groups. We hypothesized psychological distress would be positively associated with current cigarette smoking among NHPI adults and among adults from other racial/ethnic groups, and the magnitude of the association between psychological distress and current cigarette would be greater among NHPI adults compared to adults of other racial/ethnic groups.

## 2 Methods

### 2.1 Data sources

We utilized data from the 2014 general US NHIS and the 2014 NHPI-NHIS. The procedures of the two surveys were similar (e.g., used same survey instruments) with one non-institutionalized civilian adult aged ≥ 18 years randomly selected from sampled households to complete the survey (National Center for Health Statistics, 2017, National Center for Health Statistics, 2017, National Center for Health Statistics, 2016); however, the two studies employed different sampling designs. The NHIS used a random sample of US households, while the NHPI-NHIS focused exclusively on US NHPI households, resulting in a sample of 36,697 and 2,590 adult respondents for the NHIS and NHPI-NHIS, respectively.

### 2.2 Study populations

Among the respondents to the NHPI-NHIS survey, 50.8% identified as NHPI alone, and 49.2% identified as NHPI in combination with one or more other races. Adults who reported NHPI as their primary race were included in the present analysis. In the general NHIS, adults who self-identified as non-Hispanic White, non-Hispanic Black/African-American, non-Hispanic Asian (hereafter referred to as White, Black, Asian), and Hispanic were included. Adults from the NHIS sample with multiple races or no primary race selected were excluded to avoid non-identification of NHPI adults. The final analytic sample included 1,916 NHPI, 20,430 White, 4,725 Black, 2,001 Asian, and 5,710 Hispanic adults.

### 2.3 Measures

#### 2.3.1 Smoking status

*Adults currently smoking cigarettes* were defined as individuals who had smoked 100 cigarettes in their lifetime and reported they smoked “every day” or “on some days” at the time of the survey. Individuals who used both cigarette/e-cigarette or e-cigarette alone were not included in the analysis. *Adults not currently smoking cigarettes* included adults who never smoked cigarettes and those who formerly smoked cigarettes, i.e., smoked at least 100 cigarettes in their lifetime but reported they no longer smoked at the time of the survey. Those who reported they never smoked or formerly smoked were combined as the reference group for those who reported currently smoking cigarettes, as the focus of the analysis was on the association between current psychological distress and current cigarette smoking. Moreover, NHIS assessed psychological distress within the past 30 days. As such, examining the association of psychological distress with never smoked or with former smoking status would have resulted in an inappropriate retrodiction.

We excluded adults who were using e-cigarettes alone (n = 1,402) and those concomitantly smoking cigarettes due to their small numbers in certain racial/ethnic populations (e.g., for Asians n = 30), which would prevent us from comparing across subgroups (Narcisse et al., 2020).

#### 2.3.2 Psychological distress

Psychological distress was the primary independent variable of interest. This variable was measured using the Kessler 6 (K6) scale, a six-item questionnaire asking participants to rate levels of depression or sadness, nervousness, hopelessness, restlessness or fidgetiness, worthlessness, and feeling everything was an effort in the past 30 days (Kessler et al., 2002, Kessler et al., 2003). Responses for each question ranged from “none at the time” (coded 0) to “all the time” (coded 4), and when summed, the K6 yielded a score ranging between 0 and 24. The summative scale demonstrated excellent internal consistency and reliability (Cronbach's alpha = 0.86), and the summative scores were categorized to represent various levels of psychological distress: none (K6 score of 0), low (K6 score of 1–2), moderate (K6 score of 3–5), high (K6 score of 6–11), and very high (K6 score of 11–24) (Cunningham et al., 2015, Kobau et al., 2014, Zhao et al., 2009, Pratt, 2009, Park et al., 2017).

#### 2.3.3 Other covariates

Putative potential confounders of the association between psychological distress and cigarette smoking were considered in regression models based on previous studies (Park et al., 2017, Weinberger, 2020, Kaminska and Lynn, 2017). Variables employed in the analysis included: biological sex (*males or females*); age (*18*–*24, 25*–*34, 35*–*44, 45*–*54, 55*–*64, and ≥ 65 years*); marital status (*single, never married, divorced, separated or widowed; married/living in cohabitation*); education (*< high school, high school/graduate-equivalent degree, > high school*); employed for at least one week in the last year (*yes/no*); poverty (*< 100% of the federal poverty line [FPL], ≥ 100% of the FPL*), born in the US (*yes/no*), and has a usual source of health care (*yes/no*).

### 2.4 Analytic methods

#### 2.4.1 Survey-based cross-race/ethnicity comparisons

Because of the distinct sampling designs of the NHPI-NHIS and the general NHIS, we applied Kaminska and Lynn's technique (Rao and Scott, 1984) to combine data from NHPI-NHIS and the general NHIS. This technique involved developing stratum indicators, primary sampling unit indicators, and population scaling of sampling weights. Analyses were weighted using the sampling weights developed through this technique for comparative survey analysis (Rao and Scott, 1984).

#### 2.4.2 Descriptive and regression analyses

For descriptive analysis of current cigarette smoking and level of psychological distress, we computed weighted percentages and 95% confidence intervals (CI). We used the Rao-Scott Chi-Square test of independence (Sharpe, 2015) for unadjusted associations between race/ethnicity and current cigarette smoking and for unadjusted associations between race/ethnicity and psychological distress. *Post hoc* pairwise comparison tests with Bonferroni correction were used to reduce the probability of a type I error when comparisons were made between NHPIs and other racial/ethnic groups (StataCorp, 2015). We then used the Rao-Scott Chi-Square test of independence (Sharpe, 2015) for unadjusted associations between current cigarette smoking status and psychological distress within each race/ethnicity.

For each racial/ethnic group, we estimated multivariable logistic regression models reporting adjusted odds ratios (OR) to measure associations between psychological distress and current cigarette smoking.

The models were adjusted for sex, age, poverty levels, education, employment, marital status, nativity, and usual source of care. To assess multicollinearity among independent variables, we conducted the variance inflator factor (VIF). After conducting the logistic regression models, we used the Bonferroni adjusted Wald test to determine whether the adjusted associations found between psychological distress and current smoking status in the NHPI population were statistically different than the same associations found in the other racial/ethnic groups.

All statistical analyses were conducted using Stata/SE 15.1 (Sung et al., 2011). Statistical significance was set at an alpha level of 0.05.

This analysis was approved by the University of Arkansas for Medical Sciences Institutional Review Board (#206591).

## 3 Results

### 3.1 Current cigarette smoking status by race/ethnicity

Black adults had the highest current smoking rate at 13.3%, followed by White (9.7%), NHPI (8.8%), Hispanic (7.3%), and Asian (6.7%) adults. The prevalence of current cigarette smoking between NHPI and Black adults was significantly different, with more Black adults currently smoking than NHPI adults. See Table 1. Sociodemographic and socioeconomic characteristics of adults currently smoking cigarettes are presented overall and by race/ethnicity in Appendix A, Table A1.

**Table 1:** Current Cigarette Smoking and Psychological Distress Category among All Study Subjects, by Racial/Ethnic Group.^†^

Adults from Racial/Ethnic Groups	Non-Hispanic NHPI (n = 1,916)	Non-Hispanic White (n = 20,430)	Non-Hispanic Black (n = 4,725)	Non-Hispanic Asian (n = 2,001)	Hispanic (n = 5,710)
Current Cigarette Smoking	% (95% CI)	% (95% CI)	% (95% CI)	% (95% CI)	% (95% CI)
Yes	8.8 (7.0, 11.1)	9.7 (9.1, 10.4)	**13.3 (12.1, 14.6)**	6.7 (5.2, 8.5)	7.3 (6.6, 8.1)
No	91.2 (88.9, 93.0)	90.3 (89.6, 90.9)	**86.7 (85.4, 87.9)**	93.3 (91.5, 94.8)	92.7 (91.9, 93.4)
	Rao-Scott Chi-Square test: F (5.99, 1957.91) = 83.99, p < 0.001				
Psychological Distress Category	% (95% CI)	% (95% CI)	% (95% CI)	% (95% CI)	% (95% CI)
No Distress (K6 = 0)	45.3 (41.7, 48.9)	47.2 (46.0, 48.3)	**50.7 (48.6, 52.9)**	**56.4 (53.3, 59.4)**	**51.9 (50.1, 53.7)**
Low Distress (K6 = 1–2)	20.9 (18.0, 24.1)	22.0 (21.1, 22.8)	16.7 (15.2, 18.3)	17.3 (15.4, 19.5)	17.0 (15.8, 18.2)
Moderate Distress (K6 = 3–5)	13.3 (11.4, 15.4)	15.9 (15.1, 16.6)	14.7 (13.4, 16.2)	14.4 (12.2, 16.9)	13.5 (12.4, 14.6)
High Distress (K6 = 6–10)	13.2 (11.0, 15.8)	10.3 (9.7, 11.0)	11.9 (10.7, 13.2)	**8.7 (7.2, 10.5)**	10.9 (9.9, 12.0)
Very High Distress (K6 = 11–24)	7.3 (6.2, 8.6)	**4.7 (4.3, 5.1)**	6.0 (5.1, 7.0)	**3.2 (2.3, 4.5)**	6.7 (5.9, 7.7)
	Rao-Scott Chi-Square test: F (11.81, 3861.26) = 10.87, p < 0.001				

Data Source: 2014 NHIS and NHPI-NHIS.

Note: ^†^ Bolded estimates (weighted percentages and 95% Confidence Intervals) indicate percentages significantly different from NHPIs percentages.

n = unweighted number of respondents in each racial/ethnic group (excluding e-cigarette users and other racial groups), CI = Confidence Interval Level, K6 = Kessler 6, NHIS = National Health Interview Survey, NHPI = Native Hawaiian and Pacific Islander.

### 3.2 Level of psychological distress by race/ethnicity

NHPIs had the highest prevalence of very high psychological distress (7.3%), followed by Hispanic (6.7%), Black (6.0%), White (4.7%), and Asian (3.2%) adults. The proportion of NHPI with very high psychological distress was significantly higher than White and Asian adults. NHPI also had the highest prevalence of high psychological distress (13.2%), followed by Black (11.9%), Hispanic (10.9%), White (10.3%), and Asian (8.7%) adults. The proportion of NHPI with high psychological distress was significantly higher than Asian adults. Asian adults had the highest prevalence of no psychological distress (56.4%), followed by Hispanic (51.9%), Black (50.7%), White (47.2%), and NHPI (45.3%) adults. The prevalence of no psychological distress among NHPI adults was significantly lower than among Asian, Hispanic, and Black adults.

### 3.3 Psychological distress and current smoking by race/ethnicity

Across each of the racial/ethnic groups, there was a significant positive gradient in the prevalence of current cigarette smoking and the level of psychological distress. The current cigarette smoking prevalence was the lowest among those with no psychological distress and highest among those with very high psychological distress, except for Asian adults. For Asian adults, the highest prevalence of cigarette smoking was among those with high rather than very high psychological distress. See Table 2 for unadjusted associations between psychological distress and current smoking among each racial/ethnic group.

**Table 2:** Current Cigarette Smoking Prevalence for Psychological Distress Categories among Adults Who Currently Smoke, by Racial/Ethnic Group.^†^

	Non-Hispanic NHPI (n = 177)	Non-Hispanic White (n = 2,062)	Non-Hispanic Black (n = 721)	Non-Hispanic Asian (n = 129)	Hispanic (n = 474)
Psychological Distress Category	% (95% CI)	% (95% CI)	% (95% CI)	% (95% CI)	% (95% CI)
No Distress (K6 = 0)	5.6 (4.4, 7.3)	8.4 (7.7, 9.2)	11.9 (10.4, 13.6)	6.4 (4.9, 8.5)	6.2 (5.3, 7.3)
Low Distress (K6 = 1–2)	7.4 (4.3, 12.5)	8.7 (7.6, 10.0)	12.8 (9.8, 16.5)	2.9 (1.7, 4.9)	5.9 (4.5, 7.6)
Moderate Distress (K6 = 3–5)	10.4 (5.1,20.0)	9.8 (8.5, 11.3)	14.3 (11.6, 17.5)	6.6 (2.2, 18.4)	10.8 (8.3, 14.1)
High Distress (K6 = 6–10)	13.8 (7.2, 24.7)	14.6 (11.1, 18.9)	15.8 (12.2, 20.3)	14.8 (8.3, 24.9)	8.7 (6.3, 11.9)
Very High Distress (K6 = 11–24)	22.3 (11.6, 38.6)	18.6 (15.5, 22.3)	24.0 (18.3, 30.7)	7.3 (2.3, 21.3)	13.0 (9.1, 18.4)
Rao-Scott Chi-Square test	F _(3.45, 93.22)_ = 4.79, *p* < 0.01	F _(2.93, 878.49)_ = 13.68, *p* < 0.001	F _(3.90, 1083.70)_ = 5.49, *p* < 0.001	F _(2.76, 709.46)_ = 2.64, *p* = 0.053	F _(3.86, 1065.95)_ = 6.60, *p* < 0.001

Data Source: 2014 NHIS and NHPI-NHIS.

Note: ^†^ Adults are individuals aged 18 years and older.

n = unweighted number of cigarette smokers in each racial/ethnic group (excluding e-cigarette users and adults other racial groups), K6 = Kessler 6, NHIS = National Health Interview Survey, NHPI = Native Hawaiian and Pacific Islander, CI = Confidence Interval Level.

### 3.4 Psychological distress and current cigarette smoking

There was no multicollinearity among the independent variables in the regression models (VIF varied between a minimum of 1.04 to a maximum of 1.29, with a mean VIF of 1.13). NHPI with high and very high psychological distress were more than twice as likely to be current cigarette smokers than NHPI with no psychological distress (OR: 2.42, CI: 1.21–4.85 & OR: 2.86, CI: 1.47–5.55, respectively). Very high psychological distress was also associated with significantly greater odds of current cigarette smoking among White (OR: 1.68, CI: 1.30–2.18), Black (OR: 1.85; CI: 1.26–2.70), and Hispanic (OR: 2.35; CI: 1.44–3.83) but not among Asian adults. Hispanic adults with moderate distress (OR: 1.81, CI: 1.22–2.67) and Asian adults (OR: 3.36, CI: 1.48–7.63) and Non-Hispanic White (OR: 1.50, CI: 1.03–2.19) adults with high distress were also significantly more likely to currently smoke than their counterparts with no distress. See Table 3.

**Table 3:** Associations between Psychological Distress Categories and Current Cigarette Smoking among Adults Who Currently Smoke, by Racial/Ethnic Group.^†^

	Non-Hispanic NHPI	Non-Hispanic White	Non-Hispanic Black	Non-Hispanic Asian	Hispanic
Psychological Distress Categories					
No Distress (ref) (K6 = 0)	–	–	–	–	–
Low Distress (K6 = 1–2)	1.29 (0.69, 2.43)	1.05 (0.87, 1.26)	1.08 (0.75, 1.58)	0.45* (0.23, 0.90)	1.06( 0.74, 1.52)
Moderate Distress (K6 = 3–5)	1.78 (0.68, 4.64)	1.13 (0.92, 1.39)	1.33 (0.98, 1.80)	1.060 (.39, 2.89)	1.81**( 1.22, 2.67)
High Distress (K6 = 6–10)	2.42* (1.21, 4.85)	1.50* (1.03, 2.19)	1.31 (0.90, 1.91)	3.36** (1.48, 7.63)	1.54( 1.00, 2.38)
Very High Distress (K6 = 11–24)	2.86** (1.47, 5.55)	1.68*** (1.30, 2.18)	1.85** (1.26, 2.70)	1.09 (0.25, 4.80)	2.35**( 1.44, 3.83)

Data source: 2014 NHIS and NHPI-NHIS.

Notes: ^†^ Models adjusted for sex, age, poverty levels, education, employment, marital status, born in the US, and usual source of care. Adjusted Odds-Ratios and Confidence Intervals (95% CI) are weighted to account for sampling design. Adults are individuals aged 18 years and older.

*** p < 0.001, ** p < 0.01, * p < 0.05 for within racial/ethnic group differences. No significant differences between groups were found.

K6 = Kessler 6, NHPI = Native Hawaiian and Pacific Islander, REF = Reference Group.

When comparing racial and ethnic groups, the adjusted Wald test did not show any significant differences in the magnitude of the association between psychological distress and current cigarette smoking among NHPI adults relative to adults from the other racial/ethnic groups.

## 4 Discussion

We sought to determine if there was an association between psychological distress levels and current cigarette smoking among NHPI adults, as has been found for adults from other racial/ethnic groups (Park et al., 2017, Carter et al., 2014, Tomioka et al., 2021, Leung et al., 2011, Kiviniemi et al., 2011, Hickman et al., 2014, Choi and DiNitto, 2011, Kassel et al., 2003), and to determine if the magnitude of the association was greater among NHPI adults compared to the same association found among adults of other racial/ethnic groups. Current cigarette smoking was significantly lower among NHPI adults compared to Black adults but not different compared to White, Asian, and Hispanic adults. A significantly lower percentage of NHPI adults reported having no psychological distress compared to Black, Asian, and Hispanic adults, but NHPI adults were significantly more likely to report higher levels of psychological distress compared to White and Asian adults. The significant differences in current cigarette smoking and in psychological distress levels between NHPI adults and Asian adults further supports the need to disaggregate health-related data between those of various racial/ethnic groups to avoid masking significant differences found in this study, among others (Mattingly et al., 2020).

As hypothesized, we found a significant association between psychological distress levels and current cigarette smoking among NHPI adults after adjusting for other factors associated with current cigarette smoking, a finding previously unavailable in the literature. We also confirmed previous research showing the same association among adults from other racial/ethnic groups (Goodwin, 2017, Fluharty et al., 2017, Smith et al., 2014). This finding does support the use of smoking interventions culturally tailored for NHPI adults who currently smoke cigarettes, as such tailoring has been shown to be effective among American Indian individuals (Kim et al., 2015, Choi et al., 2016, Haddad et al., 2017, de Dios et al., 2019, Palmer et al., 2014). One such study reported community-based organizations played a crucial role in conducting tobacco control programs for NHPI communities and were successful in reducing tobacco-related disparities among NHPI adults as compared to adults of other races/ethnicities (Liu et al., 2013). However, compared to non-culturally-tailored interventions, cessation rates for culturally tailored interventions were no different for African American individuals and Chinese American individuals (Yalcin et al., 2014). Nevertheless, culturally-tailored interventions were significantly more acceptable to these groups (Yalcin et al., 2014), suggesting cultural-tailoring may increase participation in cessation interventions. Although the goal of such an intervention may be to increase cessation rates, participation by targeted racial/ethnic groups is critical for the cessation intervention to even have a chance to affect cessation rates.

Addressing psychological distress in smoking cessation treatment has also been shown to be effective (Veilleux, 2019). However, to our knowledge, no studies have assessed the effect of culturally-tailored smoking interventions that also address psychological distress on cessation rates. Thus, such interventions for NHPI adults and for adults of other racial/ethnic groups should be developed and tested in future research.

We did not find support for our hypothesis that the magnitude of the association would be significantly greater among NHPI adults compared to adults of other racial/ethnic groups. However, this finding suggests high and very high levels of psychological distress are associated with smoking regardless of race/ethnicity (except among Asian adults); in other words, race/ethnicity does not moderate the association between psychological distress and smoking. Future studies should include additional factors that may affect smoking and those considered in the present analysis to further explain the variation in current cigarette smoking. Although this research did not detect significant differences in smoking rates or in the odds of smoking given varying levels of psychological distress between NHPI and Asian adults, we did find that NHPI adults have significantly higher levels of psychological distress. This provides additional support for the need to disaggregate health-related data of NHPI and Asian adults.

## 5 Strengths and limitations

This study presents some limitations. First, self-reporting of current cigarette smoking and level of psychological distress is subject to courtesy bias; thus, estimates presented in this study could be inflated or deflated. Second, we report the association between psychological distress and current cigarette smoking in our study; however, this association can be complex and likely bidirectional. Although several theoretical and empirical models have examined the conceptual pathways between psychological distress and smoking (Tomioka et al., 2021, Leung et al., 2011, Dube et al., 2009, Spears et al., 2019), those models did not attempt to disentangle the moderating effect of race/ethnicity. Lastly, despite evidence of the relationship between e-cigarette and current cigarette smoking and of the relationship between e-cigarettes and psychological distress [68], this study examines the association between psychological distress and current cigarette smoking only (not e-cigarettes or other tobacco products). However, results on the relationship between psychological distress and current e-cigarette use among NHPI adults compared to adults of other racial/ethnic groups is available in the literature (Weinberger, 2020). Despite these limitations, this is the first US population-based study to examine the relationship between current cigarette smoking and psychological distress levels among a large, nationally representative sample including NHPI adults and to compare the magnitude of that association between NHPI adults and adults from other racial/ethnic groups.

## 6 Conclusions and future research

We found that psychological stress is associated with current cigarette smoking in NHPI adults but not significantly more or less than adults from other racial/ethnic groups. Smoking cessation interventions should include content to help adults cope with higher levels of psychological distress in ways other than cigarette smoking. Given our finding that psychological stress levels alone differ significantly between NHPI adults and adults from some other racial/ethnic groups, future research should explore reasons for high/very high levels of psychological distress among adults from each racial/ethnic group. This may guide development of upstream smoking prevention interventions to include unique underlying reasons for high/very high levels of psychological distress.

### CRediT authorship contribution statement

**Marie-Rachelle Narcisse:** Conceptualization, Methodology, Software, Validation, Formal analysis, Data curation, Writing – original draft, Visualization, Supervision, Project administration. **Sumit K. Shah:** Writing – original draft, Writing – review & editing, Project administration. **Holly C. Felix:** Conceptualization, Methodology, Writing – original draft, Writing – review & editing, Visualization, Supervision, Project administration. **Page D. Dobbs:** Conceptualization, Writing – review & editing. **Pearl A. McElfish:** Conceptualization, Methodology, Resources, Data curation, Writing – original draft, Writing – review & editing, Supervision, Project administration, Funding acquisition.

## Declaration of competing interest

The authors declare that they have no known competing financial interests or personal relationships that could have appeared to influence the work reported in this paper.

## Figures and Tables

**Table A1:** Population Characteristics of Adults Aged ≥ 18 Who Smoke Cigarettes, by Racial/Ethnici Group: Weighted Proportions and Confidence Intervals (95% CI).

	**Non-Hispanic NHPI**	**Non-Hispanic White**	**Non-Hispanic Black**	**Non-Hispanic Asian**	**Hispanic**
**Characteristics**	(n = 177)	(n = 2,062)	(n = 721)	(n = 129)	(n = 474)
**Sex** F_(2.86, 928.10)_ = 12.40; p < 0.001^†^					
Female	39.5 (29.0,51.1)	48.9 (45.5,52.3)	41.2 (36.5,45.9)	29.8 (19.5,42.5)	30.3 (25.8,35.1)
Male	60.5 (48.9,71.0)	51.1 (47.7,54.5)	58.8 (54.1,63.5)	70.2 (57.5,80.5)	69.7 (64.9,74.2)
**Age** F_(11.68, 3796.00)_ = 3.78; p < 0.001^†^					
18–24	**15.4 (7.7,28.5)**	8.8 (5.8,13.1)	9.3 (6.4,13.1)	**6.0 (1.6,19.4)**	11.8 (7.5,18.1)
25–34	17.8 (11.5,26.7)	15.2 (13.2,17.5)	23.2 (18.9,28.2)	15.0 (8.8,24.3)	25.1 (19.9,31.1)
35–44	25.8 (15.9,38.8)	18.3 (16.0,20.9)	19.1 (15.7,23.2)	34.5 (22.1,49.3)	21.0 (16.6,26.3)
45–54	25.9 (17.1,37.3)	21.7 (19.1,24.5)	20.5 (17.2,24.2)	24.3 (14.8,37.1)	25.2 (20.7,30.2)
55–64	8.5 (4.8,14.6)	21.4 (18.8,24.2)	18.1 (14.9,21.8)	11.0 (6.6,18.0)	11.9 (8.8,15.8)
65+	6.6 (3.8,11.2)	14.6 (12.7,16.8)	9.8 (7.7,12.3)	**9.3 (4.9,17.0)**	5.0 (3.5,7.3)
**Federal Poverty Index (FPL)** F_(8.40, 2730.72)_ = 9.95; p < 0.001^†^					
< 100% FPL	37.7 (26.3,50.7)	19.2 (16.8,21.9)	38.9 (34.5,43.4)	16.9 (9.8,27.8)	29.6 (24.5,35.3)
≥ 100% FPL	62.3 (49.3,73.7)	80.8 (78.1,83.2)	61.1 (56.6,65.5)	83.1 (72.2,90.2)	70.4 (64.7,75.5)
**Education** F_(5.67, 1844.20)_ = 11.57; p < 0.001^†^					
Less than HS/GED	**18.3 (9.1,33.4)**	17.0 (14.8,19.5)	25.2 (21.3,29.5)	14.5 (8.1,24.7)	40.4 (34.7,46.3)
HS/GED	53.9 (41.9,65.5)	37.5 (34.4,40.7)	41.0 (35.4,46.7)	33.5 (20.9,49.0)	32.9 (27.5,38.9)
Above HS/GED	27.7 (20.0,37.0)	45.5 (41.8,49.2)	33.9 (29.3,38.8)	52.0 (38.9,64.9)	26.7 (21.1,33.2)
**Employment Status** F_(3.06, 992.88)_ = 6.97; p < 0.001^†^					
Employed	62.0 (49.7,72.9)	56.6 (53.1,60.0)	50.0 (45.0,55.1)	69.9 (58.2,79.5)	66.8 (61.5,71.6)
Unemployed	38.0 (27.1,50.3)	43.4 (40.0,46.9)	50.0 (44.9,55.0)	30.1 (20.5,41.8)	33.2 (28.4,38.5)
**Marital Status** F_(3.07, 997.21)_ = 11.21; p < 0.001^†^					
Single	40.9 (30.5,52.2)	44.4 (40.8,48.0)	63.0 (58.1,67.6)	37.6 (26.2,50.7)	48.7 (42.1,55.4)
Married/Cohabitation	59.1 (47.8,69.5)	55.6 (52.0,59.2)	37.0 (32.4,41.9)	62.4 (49.3,73.8)	51.3 (44.6,57.9)
**Nativity** (Born in the US) F_(3.03, 986.37)_ = 186.31; p < 0.001^†^					
No	32.1 (23.1,42.8)	5.9 (4.4,7.9)	3.7 (2.3,6.0)	74.6 (62.9,83.6)	53.0 (47.0,58.9)
Yes	67.9 (57.2,76.9)	94.1 (92.1,95.6)	96.3 (94.0,97.7)	25.4 (16.4,37.1)	47.0 (41.1,53.0)
**Usual Source of Care** F_(3.03, 986.37)_ = 186.31; p < 0.001^†^					
No	21.7 (14.5,31.2)	17.8 (15.5,20.3)	23.0 (18.6,28.1)	18.2 (10.9,28.7)	33.7 (27.9,40.1)
Yes	78.3 (68.8,85.5)	82.2 (79.7,84.5)	77.0 (71.9,81.4)	81.8 (71.3,89.1)	66.3 (59.9,72.1)

Data source: 2014 NHIS and NHPI-NHIS.

Notes: ^†^ Rao-Scott χ^2^ test statistics. Bolded estimates should be interpreted with caution as RSE ≥ 30%. Sample sizes (n) are unweighted.

FPL = Federal Poverty Line, GED = General Educational Development, HS = High School, K6 = Kessler, NHPI = Native Hawaiian and Other Pacific Islanders, US = United States.
